# Perilipin 5 S155 phosphorylation by PKA is required for the control of hepatic lipid metabolism and glycemic control

**DOI:** 10.1194/jlr.RA120001126

**Published:** 2021-01-05

**Authors:** Stacey N. Keenan, William De Nardo, Jieqiong Lou, Ralf B. Schittenhelm, Magdalene K. Montgomery, James G. Granneman, Elizabeth Hinde, Matthew J. Watt

**Affiliations:** 1 Department of Physiology, University of Melbourne, Melbourne, Victoria, Australia; 2 School of Physics, University of Melbourne, Melbourne, Victoria, Australia; 3 Department of Biochemistry and Molecular Biology, Bio21 Institute, University of Melbourne, Melbourne, Victoria, Australia; 4 Monash Proteomics & Metabolomics Facility and Department of Biochemistry and Molecular Biology, Monash University, Victoria, Australia; 5 Center for Molecular Medicine and Genetics, Wayne State University, Detroit, MI, USA

**Keywords:** lipid droplets, perilipins, lipase, triglyceride, fatty acid, protein kinase A, ABHD5, α-β hydrolase domain-containing 5, ANOVA, analysis of variance, ATGL, adipose triglyceride lipase, HSL, hormone sensitive lipase, PKA, protein kinase A, MGL, monoacylglycerol lipase

## Abstract

Perilipin 5 (PLIN5) is a lipid-droplet-associated protein that coordinates intracellular lipolysis in highly oxidative tissues and is thought to regulate lipid metabolism in response to phosphorylation by protein kinase A (PKA). We sought to identify PKA phosphorylation sites in PLIN5 and assess their functional relevance in cultured cells and the livers of mice. We detected phosphorylation on S155 and identified S155 as a functionally important site for lipid metabolism. Expression of phosphorylation-defective PLIN5 S155A in *Plin5* null cells resulted in decreased rates of lipolysis and triglyceride-derived fatty acid oxidation. FLIM-FRET analysis of protein-protein interactions showed that PLIN5 S155 phosphorylation regulates PLIN5 interaction with adipose triglyceride lipase at the lipid droplet, but not with α-β hydrolase domain-containing 5. Re-expression of PLIN5 S155A in the liver of *Plin5* liver-specific null mice reduced lipolysis compared with wild-type PLIN5 re-expression, but was not associated with other changes in hepatic lipid metabolism. Furthermore, glycemic control was impaired in mice with expression of PLIN5 S155A compared with mice expressing PLIN5. Together, these studies demonstrate that PLIN5 S155 is required for PKA-mediated lipolysis and builds on the body of evidence demonstrating a critical role for PLIN5 in coordinating lipid and glucose metabolism.

Lipid droplets are intracellular organelles that provide a depot for triglyceride storage in almost all cells and are a central point for the control of cellular energy homeostasis. Lipolysis is the process of the sequential breakdown of triglycerides to liberate free fatty acids and glycerol, and this process is tightly controlled by dynamic interactions between lipid-droplet-associated proteins and posttranslational control mechanisms of these proteins. In this regard, adipose triglyceride lipase (ATGL) catalyzes the breakdown of triglyceride to diglyceride, which is then sequentially degraded by hormone sensitive lipase (HSL) and monoacylglycerol lipase (MGL), liberating a fatty acid with each reaction.

The perilipin (PLIN) family of proteins are among the most highly expressed lipid-droplet-associated proteins and their tissue expression is well described (1, 2, 3, 4, 5). PLIN5 has been the subject of considerable interest owing to its high expression in oxidative tissues such as the heart, brown adipose tissue, skeletal muscle, and the liver (6, 7, 8), its upregulation in response to metabolic perturbations such as increased dietary lipid availability and exercise training (9, 10, 11), and its demonstrated roles in regulating lipid metabolism (2, 12). In this context, PLIN5 coats the surface of lipid droplets where its primary function is to prevent excessive triglyceride lipolysis under states of energy sufficiency (13, 14, 15). The exact mechanism of inhibition is not yet definitive, but the prevailing notion is that under basal conditions PLIN5 independently binds ATGL and α-β hydrolase domain-containing 5 (ABHD5, also known as CGI-58), an activator of ATGL lipase activity, which prevents ATGL and ABHD5 from interacting and thereby suppresses lipolysis (16, 17).

Lipolysis is increased upon β-adrenergic activation, and protein kinase A (PKA) plays a pivotal role in regulating this process. A hallmark of lipolytic regulation is cAMP-mediated activation of PKA, leading to serine phosphorylation of proteins that are critical regulators of this process. For example, PKA phosphorylation on serine 563 and 660 of HSL (18, 19, 20), serine 239 of ABHD5 (21) and serine 406 of ATGL (22) increases the activity of these proteins and lipolysis. In addition, phosphorylation of several serine residues in PLIN1 increases lipolysis in adipocytes by regulating HSL lipolytic action at the lipid droplet surface (23, 24), while phosphorylation of serine 517 of PLIN1 regulates ATGL-dependent lipolysis (25).

Consistent with a role for PKA in regulating lipolysis, we and others have shown that PLIN5 is phosphorylated by PKA (13) and that phosphorylation of serine 155 is sufficient to activate PLIN5-regulated lipolysis (26, 27, 28). A new role for PLIN5 was recently identified showing that catecholamine stimulation induces PLIN5 serine 155 phosphorylation, resulting in PLIN5 nuclear translocation, where it forms transcriptional complexes with peroxisome proliferator-activated receptor-gamma coactivator-1-α (PGC-1α) and sirtuin 1 (SIRT1) to induce expression of PGC-1α target genes, ultimately increasing mitochondrial capacity (28, 29). These results suggest that PLIN5 couples catecholamine activation of PKA and lipid droplet lipolysis with transcriptional regulation of mitochondrial proteins to enhance the capacity for mitochondrial fatty acid oxidation.

It is evident that PLIN5 regulates intracellular fatty acid fluxes and may drive the reprogramming of cells to prevent cellular lipotoxicity. This study aimed to determine PKA-sensitive PLIN5 phosphorylation sites, to assess the metabolic roles of PLIN5 phosphorylation in vitro, and to ascertain whether these effects are maintained in vivo.

## Results

### Identification of PKA phosphorylation sites in PLIN5

**Fig. 1:**
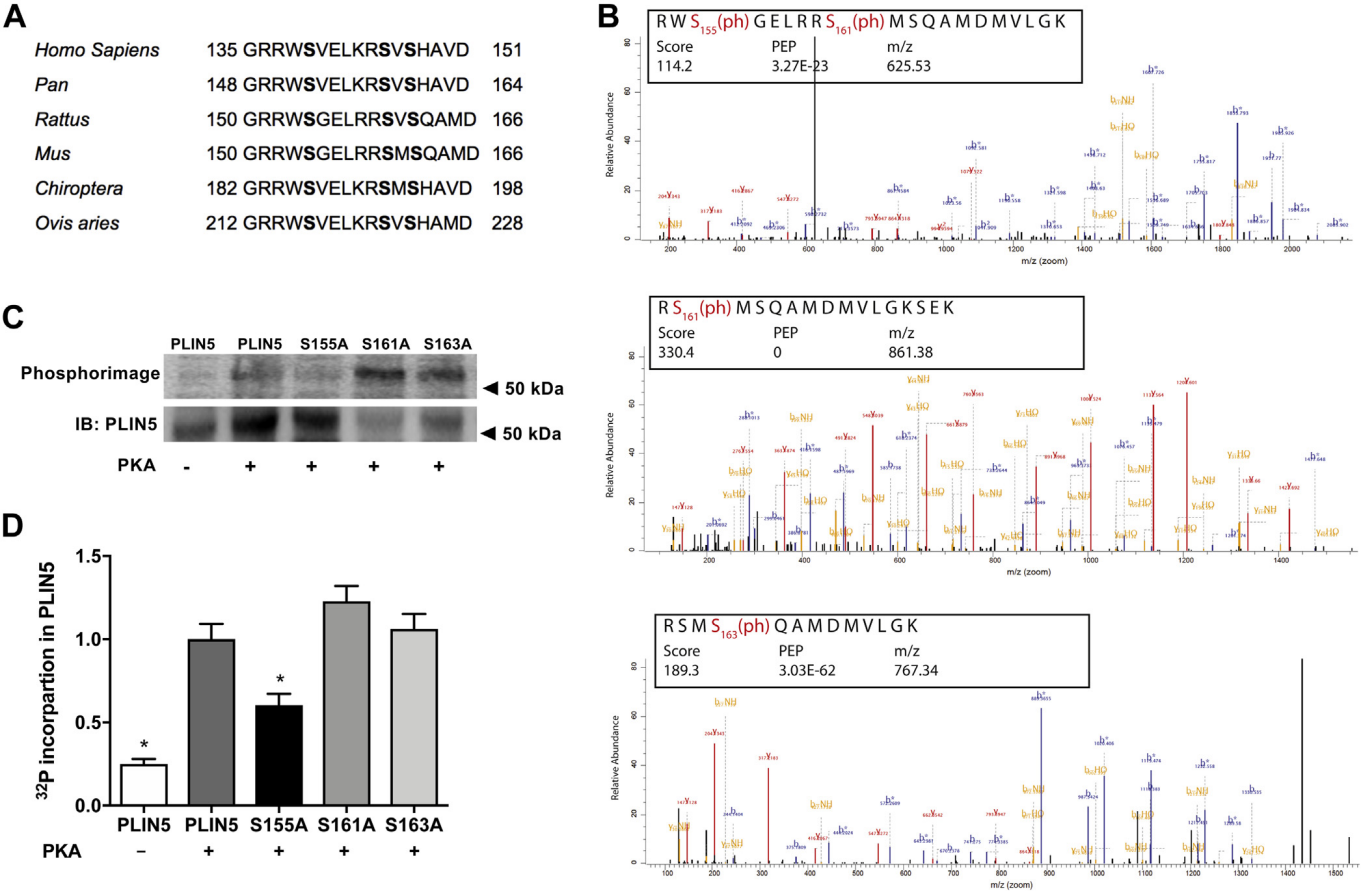
PLIN5 is a substrate for in vitro phosphorylation by PKA. A: Amino acid sequence of a portion of PLIN5 are aligned for *homo sapiens* (135–151), *pan* (148–164), *rattus* (150–166), *mus* (150–166), *chiroptera* (182–198), and *ovis aries* (212–228). The sequences show conservation at the PKA consensus sequence corresponding to murine S155 and putative sites at S161 and S163. B: Purified recombinant murine PLIN5 was incubated with PKA in vitro, the peptides were digested and phospho-peptides detected by LC-MS/MS. Data were reproduced in three independent experiments. C: Recombinant murine PLIN5 was incubated with the catalytic subunit of PKA and [γ-^32^P]ATP followed by SDS-PAGE, transfer to a PVDF membrane and autoradiography. Representative autoradiograph (above) and PLIN5 immunoblot (below). D: Quantification of [γ-^32^P]ATP incorporation into PLIN5; *n* = 2 per group. For panel (D), one-way ANOVA with Bonferroni post hoc analysis was performed. ∗*P* < 0.05 versus PLIN5 + PKA.

Analysis of the predicted amino acid sequence of murine PLIN5 identified putative PKA phosphorylation sites (NetPhos 3.1) at S155, S161, and S163, which were highly conserved in other vertebrates (Fig. 1A). To confirm that PLIN5 was a PKA substrate (26, 27, 29), murine *Plin5* recombinant protein purified from *Escherichia coli* was incubated with the catalytic subunit of PKA. The proteins were digested with trypsin and analyzed by high-resolution mass spectrometry. This analysis confirmed S155, S161, and S163 as *in vitro* PKA-responsive phosphorylation sites of *Plin5* (Fig. 1B).

A complimentary approach was employed to determine whether these serine residues on PLIN5 were substrates for PKA. Recombinant murine PLIN5 and PLIN5 harboring phosphorylation-defective serine to alanine mutations, including S155A, S161A, and S163A, were incubated with the catalytic subunit of PKA and [γ-^32^P] ATP in vitro, and the incorporation of radioactive phosphate into PLIN5 was monitored by phosphorimaging analysis. As shown in the autoradiograph (Fig. 1C) and by quantification (Fig. 1D), PLIN5-S155A was a poor substrate for PKA, whereas incorporation of γ-^32^P into PLIN5-S161A and PLIN5-S163A was indistinguishable from wild-type PLIN5. These data indicate that S155 is the primary phosphorylation site for PKA in vitro.

### Effects of PLIN5 S155, S161, and S163 phosphorylation on lipid metabolism in vitro

**Fig. 2:**
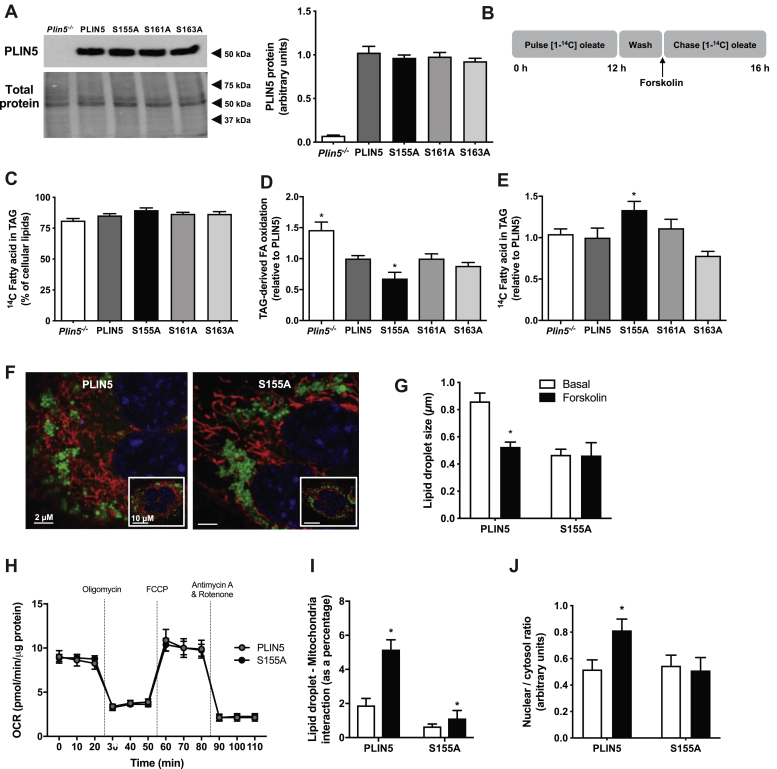
Phosphorylation of PLIN5 impacts cellular lipid metabolism. A: Protein content of PLIN5 and PLIN5 serine to alanine mutants in *Plin5*^*−/−*^ MEFs. Representative immunoblot (left) and quantification (right); *n* = 4 per group. B: “Pulse-chase” experimental design. Cells were incubated with “pulse” media containing [1-^14^C] oleate, washed, and stimulated with forskolin. Metabolism was assessed in the 4 h “chase” period. C: ^14^C fatty acid incorporated into triglyceride (TAG), presented as a percentage of total cellular lipids. D: TAG-derived fatty acid (FA) oxidation. E: ^14^C fatty acid incorporation into TAG. For panels (C–F), *n* = 6 per group from two independent experiments. F: Representative images of lipid droplets (green) and mitochondria (red) in MEFs. G: Quantification of lipid droplet size in MEFs expressing PLIN5 and PLIN5-S155A; *n* = 10 per group from two independent experiments. H: Oxygen consumption rate (OCR), measured by the Seahorse extracellular flux analyzer (A–H: *Plin5*^*−/−*^*n* = 3, PLIN5 *n* = 3, S155A *n* = 3, S161A *n* = 3, S161A *n* = 3, performed in triplicate). ∗*P* < 0.05 versus PLIN5. I: Quantification of lipid droplet-mitochondria interactions in MEFs; *n* = 10 per group from 2 independent experiments. J: Quantitation of the nucleocytoplasmic ratio of PLIN5 or PLIN5-S155A before and after forskolin administration. For panels (A, C–E), one-way ANOVA with Bonferroni post hoc analysis was performed. For panels (G–J), two-way ANOVA with Bonferroni post hoc analysis was performed. ∗*P* < 0.05 versus PLIN5.

To determine the functional relevance of these PLIN5 phosphorylation sites, wild-type PLIN5 and PLIN5-S155A, PLIN5-S161A, and PLIN5-S163A mutants were transfected into *Plin5*^*−/−*^ murine embryonic fibroblasts (MEFs) and lipid metabolism was assessed by radiometric techniques. Similar expression of PLIN5 and PLIN5 mutants was confirmed by immunoblot analysis (Fig. 2A). Initial experiments were conducted in cells under basal conditions (i.e., non-PKA stimulated). Consistent with the notion that the serine sites are PKA substrates, we observed no significant differences between groups for fatty acid uptake, oxidation, or storage into lipids (data not shown). MEFs were incubated in medium containing [1-^14^C] oleate for 12 h to load radiolabelled fatty acids into cells and to enrich lipid droplets with radiolabeled triglycerides (*i.e.*, pulse period), after which cells were washed, the fatty acids were removed, and the PKA activator forskolin was added to the culture medium, thereby facilitating assessment of lipid-droplet-derived fatty acid metabolism under stimulated conditions (Fig. 2B). On average, 85.3 ± 1.5% of the radiolabeled fatty acids were stored in triglycerides, and there were no differences between groups (Fig. 2C). Expression of wild-type PLIN5 reduced triglyceride-derived fatty acid oxidation by 30% compared with *Plin5*^*−/−*^, reaffirming PLIN5's inhibitory role on lipolysis. The oxidation of fatty acids derived from intracellular triglycerides was reduced in MEFs expressing PLIN5-S155A compared with PLIN5, and there were no differences between PLIN5, PLIN5-S161A, and PLIN5-S163A (Fig. 2D). Similarly, the amount of ^14^C-oleate remaining in triglyceride after the 4 h “chase” period was increased in PLIN5-S155A MEFs compared with PLIN5, indicating a reduction in lipolysis (Fig. 2E). There was no difference in ^14^C-oleate incorporation into cholesterol esters between PLIN5 and PLIN5 S-A mutants, indicating no effect on cholesterol ester hydrolase activity (PLIN5: 1.0 ± 0.1, S155A: 1.2 ± 0.2, S161A: 1.0 ± 0.2, S163A: 1.2 ± 0.2 arbitrary units, *n* = 6 per group). As there were no significant effects of mutating S161 or S163 on these metabolic processes, we focused on the physiological impact of the S155A mutation in subsequent studies. In agreement with the functional assessment of fatty acid metabolism, the average lipid droplet size decreased significantly in PLIN5 wild-type cells upon stimulation of PKA activity with forskolin, indicating activation of lipolysis and release of free fatty acids (Fig. 2F, G). Conversely, cells expressing the PLIN5-S155A mutant had smaller lipid droplets under basal conditions and lipid droplet size remained unaltered following forskolin administration, further highlighting impairments in PKA activation of lipolysis (Fig. 2F, G).

PLIN5-coated lipid droplets exist in close proximity to mitochondria (30, 31), and loss of PLIN5 reduces mitochondrial oxidative capacity (31, 32). To determine whether changes in triglyceride metabolism in PLIN5-S155A MEFs were mediated by a generalized defect in mitochondrial function, we assessed basal and FCCP-stimulated mitochondrial respiration. The S155A mutation in PLIN5 had no significant effect on basal state or maximal respiratory capacity, mirroring oxygen consumption and thereby substrate oxidation measured in PLIN5 MEFs (Fig. 2H). Previous studies indicate that PLIN5 is required to facilitate the interaction between lipid droplets and mitochondria, although this remains equivocal (13, 14) and may be tissue-specific. We used live cell imaging to assess organelle interaction in cells under basal and forskolin (i.e., PKA)-stimulated conditions. The interaction between lipid droplets and mitochondria in PLIN5 MEFs was increased ∼2.5-fold above basal upon forskolin administration, and the lipid droplet-mitochondria interaction was reduced by 66% under basal and 78% under stimulated conditions in PLIN5-S155A compared with wild-type PLIN5 cells (Fig. 2I). These data are consistent with the notion that phosphorylation of S155 is required for the interaction between lipid droplets and mitochondria, presumably to facilitate effective β-oxidation. In addition, phosphorylation of serine 155 promotes PLIN5 translocation to the nucleus to form a complex with PGC-1α and SIRT1 to induce expression of PGC-1α target genes and therefore enhance mitochondrial capacity (28, 29). Thus, we quantified the nuclear content of PLIN5 in cells expressing PLIN5 and PLIN5-S155A (Fig. 2J). PLIN5 nuclear content was increased by 60% above basal following PKA stimulation in cells expressing wild-type PLIN5 content, whereas no such increase was detected in cells expressing PLIN5-S155A. Together, these data show that PLIN5 phosphorylation of S155 is fundamental to promote interactions between the lipid droplet and mitochondria and to enhance β-oxidation.

### PLIN5 phosphorylation of S155 mediates interactions with ATGL, but not ABHD5

**Fig. 3:**
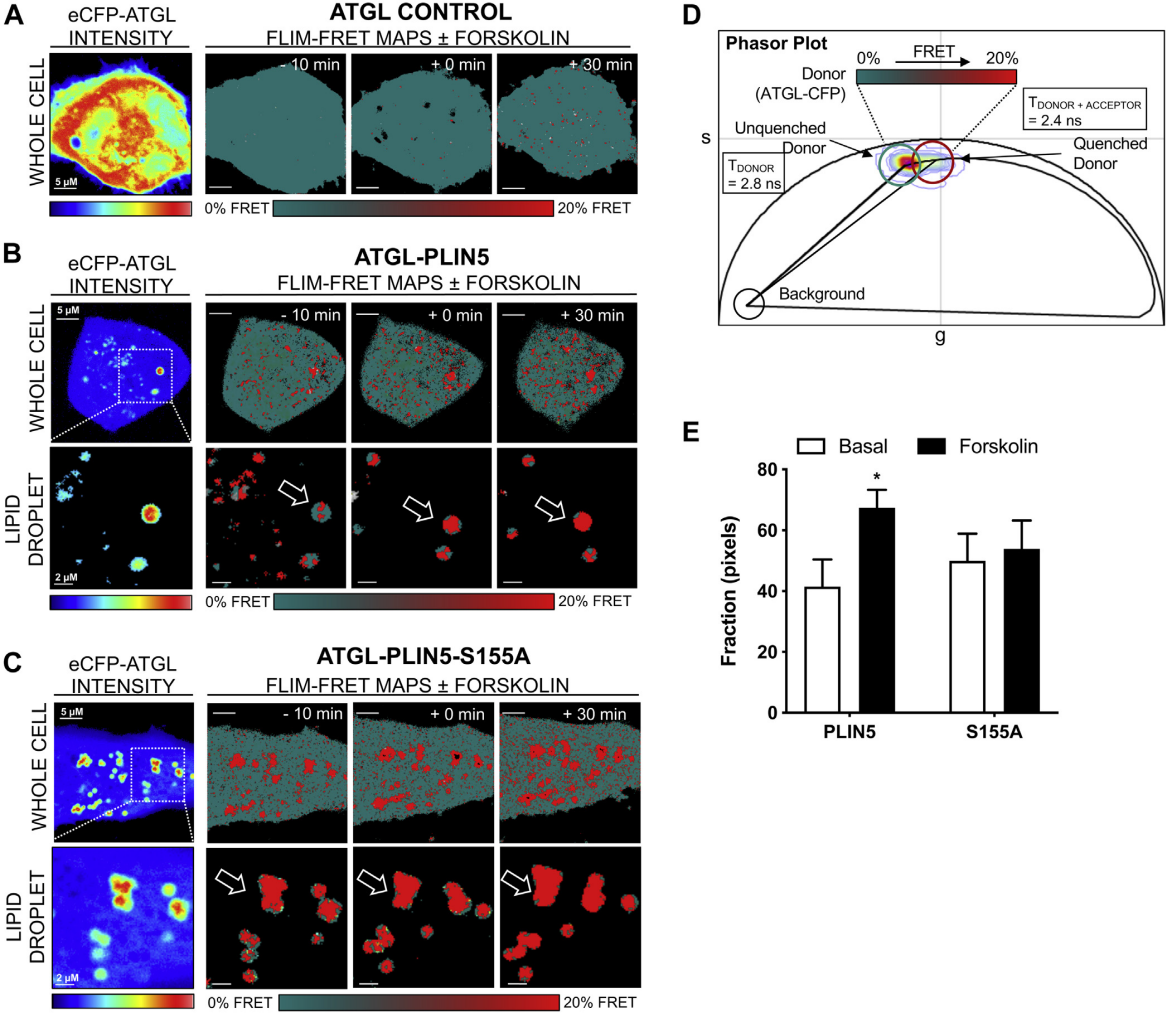
Phasor-based FLIM-FRET reveals requirement of PLIN5 phosphorylation at S155 to mediate interaction with ATGL. A: Intensity images acquired in the donor (CFP) (A: left) channel of live HeLa cells expressing ATGL-CFP in the absence (denoted ATGL control) of PLIN5-YFP or PLIN5 S155A-YFP before and after stimulation with 20 μM forskolin. Fluorescence lifetime images acquired in the donor (CFP) (A: right) channel of the different live HeLa cells pseudo-colored to report no FRET (teal pixels, 2.8 nanoseconds [ns]) versus FRET (red pixels, 2.4 ns). B, C: FRET analysis of FLIM images with an intensity threshold applied to select *only* the lipid droplets in HeLa expressing ATGL-CFP in the presence of PLIN5-YFP (B) versus S155A-YFP (C) before and after stimulation with 20 μM forskolin. D: Superimposition of the combined phasor distribution of ATGL-CFP in the absence (unquenched donor control, teal cursor) versus presence of PLIN5-YFP or S155A-YFP (FRET experiments, red cursor) with a theoretical FRET trajectory superimposed. The FRET efficiency of ATGL-PLIN5 interaction is 20%. E: Quantitation of the number of pixels undergoing FRET in FLIM images of HeLa expressing ATGL-CFP in the absence versus presence of PLIN5-YFP or PLIN5 S155A-YFP before and after 20 μM forskolin administration. Data presented as mean ± SEM for *n* = 11–13 cells from three independent experiments. For panel (E), two-way ANOVA with Bonferroni post hoc analysis was performed. ∗*P* < 0.05 basal versus forskolin.

**Fig. 4:**
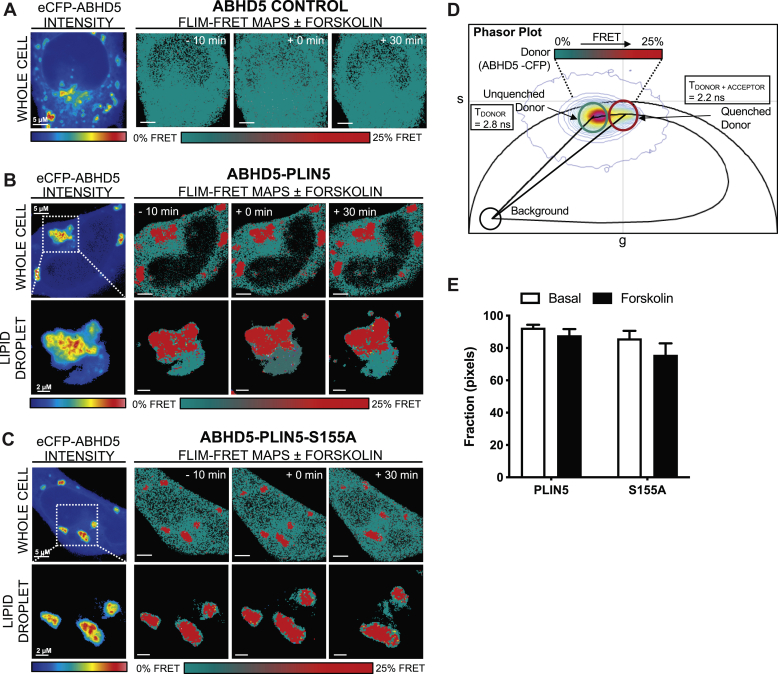
Phasor-based FLIM-FRET shows that PLIN5 phosphorylation at S155 does not mediate interactions with ABHD5. A: Intensity images acquired in the donor (CFP) (A: left) channel of live HeLa cells expressing ABHD5-CFP in the absence (denoted ABHD5 control) of PLIN5-YFP or PLIN5 S155A-YFP (denoted S155A-YFP), before and after stimulation with 20 μM forskolin. Fluorescence lifetime images acquired in the donor (CFP) (A: right) channel of the different live HeLa cells pseudo-colored to report no FRET (teal pixels, 2.8 ns) versus FRET (red pixels, 2.2 ns). B, C: FRET analysis of FLIM images with an intensity threshold applied to select *only* the lipid droplets in HeLa expressing ABHD5-CFP in the presence of PLIN5-YFP (B) versus S155A-YFP (C) before and after stimulation with 20 μM forskolin. D: Superimposition of the combined phasor distribution of ABHD5-CFP in the absence (unquenched donor control, teal cursor) versus presence of PLIN5-YFP or S155A-YFP (FRET experiments, red cursor) with a theoretical FRET trajectory superimposed. FRET efficiency of ABHD5-PLIN5 interaction is 25%. E: Quantitation of the number of pixels undergoing FRET in FLIM images of HeLa expressing ABHD5-CFP in the absence versus presence of PLIN5-YFP or PLIN5 S155A-YFP before and after 20 μM forskolin administration. Data are means ± SEM for *n* = 13–14 cells from three independent experiments. For panel (E), two-way ANOVA with Bonferroni post hoc analysis was performed.

PLIN5 interacts with key regulators of lipolysis including ATGL and ABHD5 (33). By use of the phasor approach of FLIM-FRET (for detailed methodology, see (34, 35)) we were able to investigate the impact of PLIN5 S155 phosphorylation on interactions with these key lipolytic proteins. In particular, we quantitated live cell protein interactions between ATGL-CFP or ABHD5-CFP and PLIN5-YFP or PLIN5 S155A-YFP (denoted as S155A-YFP) at the surface of lipid droplets. In each case, a phasor-based FRET analysis of the FLIM images acquired in the donor channel, ATGL-CFP (Fig. 3) or ABHD5-CFP (Fig. 4), enabled the efficiency of FRET interaction (*i.e.*, binding affinity) with PLIN5 or its phosphomutant S155A to be characterized and the frequency of this interaction to be spatially mapped within multiple cells.

In the case of ATGL, the fluorescence lifetime of ATGL-CFP (donor) in the absence of an acceptor was centered at 2.8 nanoseconds (ns), and forskolin treatment did not significantly change this baseline value (Fig. 3A, ATGL control). This indicates that forskolin treatment did not alter ATGL-CFP donor fluorescence lifetime and is therefore an appropriate baseline to quantify FRET. In contrast, in the presence of an acceptor, PLIN5-YFP versus the phospho-mutant S155A-YFP, the fluorescence lifetime of ATGL-CFP was quenched to 2.4 ns in specific locations of the cell (red pixels), and this lifetime shift was increased upon forskolin treatment in cells expressing wild-type PLIN5 but not S155A-YFP (Fig. 3B, C, E). Phasor analysis of the lifetime shift induced upon ATGL-CFP interaction with PLIN5-YFP and S155A-YFP recovered a FRET efficiency of 20% (Fig. 3D). Quantification of the fraction of pixels within each FLIM image exhibiting FRET (Fig. 3E) confirmed that forskolin stimulation induced a significant increase in the interaction between ATGL-CFP and PLIN5-YFP, but no such forskolin-induced increase was evident with S155A-YFP at the lipid droplet. This suggests that S155 phosphorylation is required for efficient PLIN5 interactions with ATGL at the surface of lipid droplets upon β-adrenergic stimulation.

In the case of ABHD5, the fluorescence lifetime of ABHD5-CFP in the absence of an acceptor was again centered at 2.8 ns, and forskolin induced a small but nonsignificant change to this baseline value (Fig. 4A, ABHD5 control). In the presence of PLIN5-YFP versus the phospho-mutant S155A-YFP, the fluorescence lifetime of ABHD5-CFP was quenched to 2.2 ns in specific locations of the cell (red pixels) (Fig. 4A), a lifetime shift that corresponds to a FRET efficiency of 25% (Fig. 4D). FRET was not significantly promoted upon forskolin treatment at the lipid droplet (Fig. 4B, C, E). This indicates that PLIN5 S155 phosphorylation is not critical for its interaction with ABHD5. Together, the studies in cells demonstrate that PLIN5 S155 phosphorylation is required to maintain lipolysis and fatty acid β-oxidation that is dependent on efficient interaction between PLIN5 and ATGL.

### Effects of PLIN5 S155 on liver lipid metabolism in mice

**Fig. 5:**
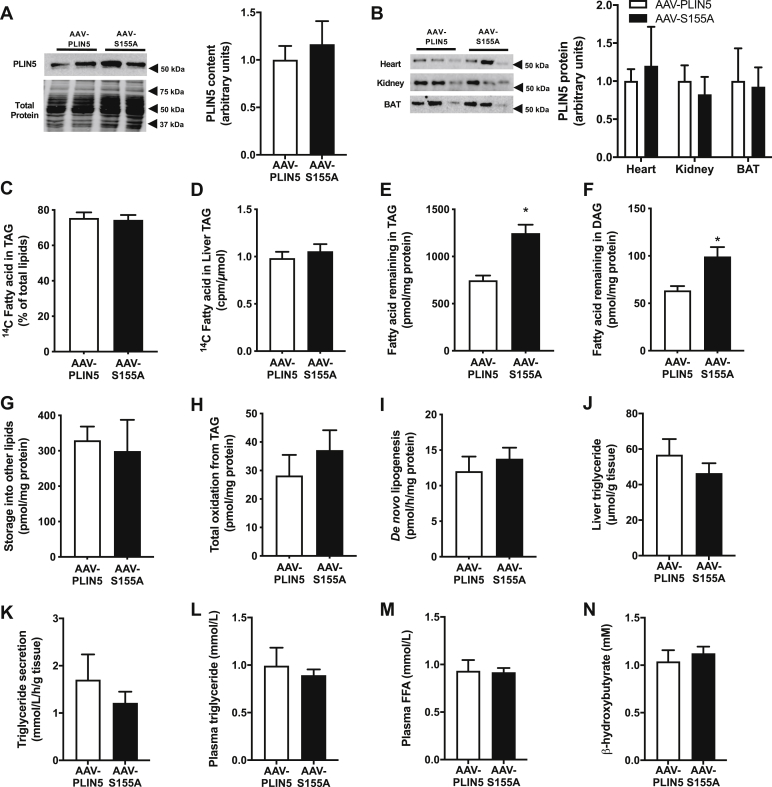
Effect of PLIN5-S155A on lipid metabolism in livers of mice. A: Representative immunoblot showing PLIN5 and PLIN5-S155A re-expression in the livers of *Plin5*^*−/−*^ mice. Quantification of immunoblot shown to the right. B: PLIN5 content in the heart, kidney, and brown (BAT) and white (WAT) adipose tissue of mice. Quantification of immunoblot shown to the right. Representative immunoblotting shows *n* = 3 per group, *n* = 7 per group for quantification. C–H: Fatty acid metabolism was assessed in precision-cut liver slices *ex vivo*. C: ^14^C-fatty acid incorporation into triglyceride (TAG) as a percentage of ^14^C-fatty acid in all lipids (at the end of the “pulse” period). Specific activity in triglycerides calculated as total cpm in triglyceride divided by moles of triglyceride (D), fatty acid remaining in TAG (E), diglycerides (DAG) (F), and all other lipids at the end of the “chase” period (G). H: Oxidation of fatty acids derived from TAG determined during the “chase” period. *De novo* lipogenesis (I), liver triglyceride content assessed by biochemical analysis (J), triglyceride secretion from precision-cut liver slices (K), plasma triglycerides in mice (L), plasma free fatty acids (FFA) (M), and plasma β-hydroxybutyrate levels in mice (N). For all panels, AAV-PLIN5 *n* = 7, AAV-S155A *n* = 7. For panels (A–N), unpaired two-tailed *t* test was performed. ∗ *P* < 0.05 AAV-PLIN5 versus AAV-S155A.

To assess the relevance of PLIN5 serine 155 phosphorylation *in vivo*, we used adeno-associated virus with an albumin promotor to express PLIN5 or PLIN5 S155A in the livers of hepatocyte-specific *Plin5* null mice (denoted herein as AAV-PLIN5 and AAV-S155A, respectively). We selected this experimental model because PLIN5 is highly expressed in the liver and is an important regulator of hepatic lipid metabolism (36). *Plin5* mRNA expression in AAV-PLIN5 and AAV-S155A was comparable with wild-type mice (*Plin5*^*LKO*^ 0.06 ± 0.03; AAV-PLIN5 0.75 ± 0.14; AAV-S155A 0.67 ± 0.15; wild-type 1.00 ± 0.20 AU, *P* < 0.05, *n* = 5 per group), and immunoblot analysis confirmed similar re-expression of PLIN5 in AAV-PLIN5 and AAV-S155A mice (Fig. 5A). AAV-mediated expression of PLIN5 in the liver was not associated with differences in PLIN5 expression in other tissues including the heart, kidney, and adipose tissues, indicating specificity of the transgene delivery (Fig. 5B).

Lipid metabolism was assessed using freshly prepared 300 μm thick precision cut liver slices obtained from AAV-PLIN5 and AAV-S155A mice. Pulse-chase experiments were performed to assess triglyceride metabolism as described for MEFs above. Briefly, liver slices were pulsed in medium containing [1–^14^C] oleate for 12 h, followed by a 6 h chase in the presence of 2 μM forskolin. The 6 h incubation period was required due to the relatively slow turnover of lipids in these preparations when compared with cultured MEFs (Fig. 2). Viability of the liver slices was assessed by measuring cellular ATP content, which averaged 43.9 ± 5.7 nmol/g and did not decline over 16 h incubation. The amount of radiolabeled fatty acids within the triglyceride pool, when expressed as a percentage of the radioactivity in all cellular lipids at the end of the pulse period, averaged 75.3 ± 3.3% and was not significantly different between groups (Fig. 5C). The specific activity of the triglyceride pool was not significantly different between groups (Fig. 5D). Consistent with the *in* *vitro* data in MEFs, the ^14^C-oleate remaining in triglycerides and diglycerides at the end of the chase period was significantly greater in AAV-S155A compared with AAV-PLIN5, which is reflective of reduced lipolysis (Fig. 5E, F). The amount of ^14^C-oleate remaining in other lipids was not different between groups, indicating specificity of PLIN5 S155A for lipolysis (Fig. 5G). Unlike the *in* *vitro* data, however, triglyceride-derived fatty acid oxidation was not significantly different between groups (Fig. 5H). Triglycerides can also be produced from *de novo* lipogenesis, which we examined using a ^14^C-glucose tracer. There were no significant differences between groups for *de novo* lipogenesis (Fig. 5I). Furthermore, liver triglyceride levels were not different between experimental groups (Fig. 5J). In light of the differences in hepatic triglyceride metabolism, without significant changes in liver triglyceride content, we next assessed rates of liver triglyceride secretion from precision-cut liver slices and plasma lipid levels in fasted mice. Neither triglyceride secretion (Fig. 5K), plasma triglycerides (Fig. 5L), plasma free fatty acids (Fig. 5M) nor plasma β-hydroxybutyrate (Fig. 5N) was significantly different between groups. Together, these data demonstrate that PLIN5 S155 is an important regulator of triglyceride metabolism in the liver but that this is insufficient to significantly alter triglycerides content or triglyceride secretion.

### Effects of PLIN5 S155A on whole-body energy balance and glycemic control in mice

**Fig. 6:**
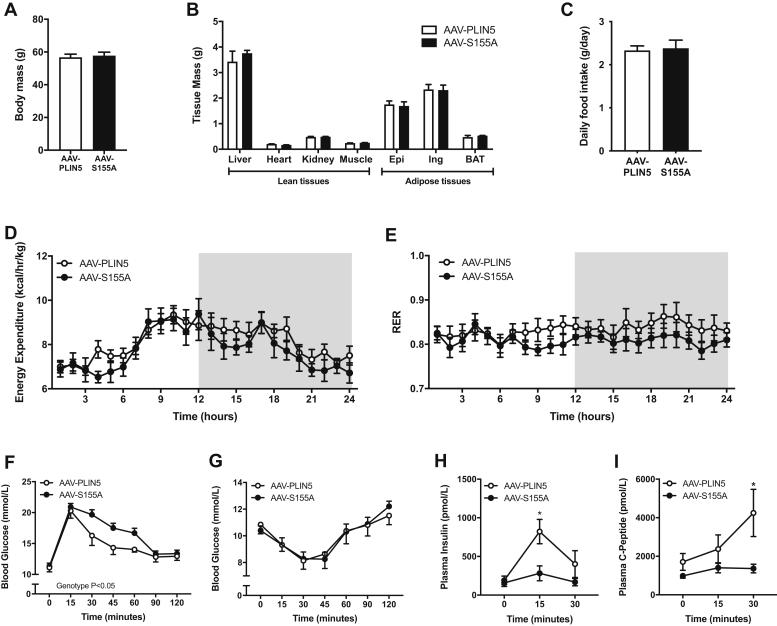
Effect of PLIN5-S155A on whole-body energy metabolism and glycemic control. Body mass of mice (A), tissue masses (B), daily food intake (C), energy expenditure (D)_,_ respiratory exchange ratio (RER) (E), oral glucose tolerance test (2 g/kg) (F), intraperitoneal insulin tolerance test (1 U/kg) (G), plasma insulin levels (H), and plasma C-peptide levels before and after glucose administration (I). For all panels, AAV-PLIN5 *n* = 7, AAV-S155A *n* = 7. For panels (A–C), unpaired two-tailed *t* test was performed. For panels (D–I), two-way ANOVA with Bonferroni post hoc analysis was performed. ∗ *P* < 0.05 AAV-PLIN5 versus AAV-S155A.

Modulating PLIN5 expression has been shown to impact whole-body energy homeostasis and glycemic control in mice (13, 26, 37), particularly in mice fed a high-fat diet. Therefore, we sought to understand whether PLIN5 S155A is important in regulating these processes. Body mass, organ weights, and fat mass were not significantly different between AAV-PLIN5 and AAV-S155A mice 12 weeks after AAV administration (Fig. 6A, B). Consistent with this result, daily food intake and whole-body energy expenditure were similar between groups (Fig. 6C, D). There was no difference in respiratory exchange ratio between AAV-PLIN5 and AAV-S155A (Fig. 6E).

We previously reported that *Plin5*^*LKO*^ mice have impaired glucose tolerance and are insulin-resistant when compared with wild-type mice and that addition of AAV-PLIN5 in *Plin5*^*LKO*^ mice restores glycemic control and insulin action (36). Consistent with this previous study, both AAV-PLIN5 and AAV-S155A administration restored glucose tolerance (glucose incremental area under the curve: *Plin5*^*LKO*^ 2242 ± 71; AAV-PLIN5 1741 ± 87; AAV-S155A 1930 ± 57 mmol/L∗120 min, *P* < 0.05, *n* = 7 per group) and insulin sensitivity in *Plin5*^*LKO*^ mice (insulin area under the curve: *Plin5*^*LKO*^ 1361 ± 53; AAV-PLIN5 1,164 ± 86; AAV-S155A 1178 ± 40 mmol/L∗120 min, *P* < 0.05, *n* = 7 per group). Hence, both AAV treatments of PLIN5 rescued the *Plin5*^*LKO*^ glucose metabolism phenotype.

We next compared AAV-PLIN5 with AAV-S155A mice only. Whole-body glycemic control, as assessed by an oral glucose tolerance test, was significantly different between groups. AAV-S155A mice were markedly glucose-intolerant when compared with AAV-PLIN5 mice (Fig. 6F). Insulin sensitivity was not significantly different between the AAV-PLIN5 and AAV-S155A mice (Fig. 6G), rather impaired glycemic control was associated with a reduction in glucose-stimulated insulin levels in AAV-S155A mice compared with AAV-PLIN5 mice (Fig. 6H). Plasma C-peptide levels were also significantly different between AAV-PLIN5 and AAV-S155A mice, indicating differences in insulin secretion between groups (Fig. 6I). Together, these results demonstrate that loss of PLIN5 S155 phosphorylation in the liver is sufficient to impair whole-body glucose tolerance and raise the intriguing possibility that this occurs through an indirect signaling mechanism that impacts pancreatic β-cell function and insulin secretion.

## Discussion

PKA activation and phosphorylation of key serine residues in regulatory proteins are a hallmark of lipolysis control. In this study, we identified S155 of PLIN5 as a key site for regulating hepatic lipid metabolism and systemic glycemic control through modulation of fatty acid flux in cells.

It is becoming increasingly clear that PLIN5 and PLIN1 exert similar roles in oxidative tissues and adipocytes, respectively, by controlling lipases and other proteins required for efficient triglyceride catabolism (23, 25, 38, 39). It is also unequivocal, at least for PLIN1, that PKA-mediated phosphorylation of serine residues is critical to induce translocation and docking of lipolytic proteins to facilitate triglyceride hydrolysis and protein-protein interactions. Under basal conditions, ABHD5 is bound to PLIN1. PKA phosphorylation of PLIN1 leads to the release of ABHD5, which allows ABHD5 stimulation of triglyceride hydrolytic activity by ATGL (23, 40, 41, 42). In addition, phosphorylation of one or more PKA sites on PLIN1 is required for the docking of HSL (43) to induce maximal lipolysis (44, 45).

With respect to PLIN5, Pollak *et al* (26) showed that PKA-induced lipolysis was substantially impaired from isolated cardiac lipid droplets enriched with PLIN5 carrying an alanine mutation at serine 155 (PLIN5 S155A), suggesting a critical role for PKA in PLIN5-regulated lipolysis. In line with this previous work in isolated lipid droplets, our studies in intact cells demonstrate that PLIN5 S155 is required to maintain fatty acid flux from intracellular triglycerides. This finding was also confirmed in liver slices *ex* *vivo*, where expression of PLIN5 S155A in *Plin5*^*−/−*^ hepatocytes led to a reduction in triglyceride breakdown when compared with hepatocytes expressing wild-type PLIN5. Concordant with our major findings, others showed that adenoviral-mediated expression of PLIN5 S155A in *Plin5*^*−/−*^ primary hepatocytes resulted in impaired glucagon (*i.e.*, PKA)-induced lipolysis compared with PLIN5 expressing hepatocytes (27). Together, these data show that akin to PLIN1's important role in regulating adipocyte lipolysis, PLIN5 plays an integral role in managing triglyceride turnover in MEFs and hepatocytes via phosphorylation of S155. Further studies will be required to demonstrate conservation of this response in other cell types where PLIN5 is highly expressed, such as cardiomyocytes, skeletal myotubes, and brown adipocytes.

The central mechanism for regulating PKA-dependent lipolysis is via the activation of ATGL and ABHD5 (46, 47, 48). Our data using the phasor approach to FLIM-FRET in live cells shows that phosphorylation of PLIN5 S155 is required for efficient PKA-mediated interaction between ATGL and PLIN5 at the surface of the lipid droplet, which is associated with decreased lipolysis and triglyceride-derived fatty acid oxidation, and thereby represents an important event in regulating lipolysis. Previous studies have shown that ABHD5 contains an allosteric binding site that regulates its interactions with PLIN proteins and provides a mode of lipolytic regulation, independent of extracellular signaling (49). This concept, shown previously by FRAP, was reaffirmed by our FLIM data demonstrating PLIN5 had a greater affinity to bind to ABHD5, than ATGL (ABHD5: 25% FRET efficiency vs. ATGL: 20% FRET efficiency). It is important to note, however, that the ATGL-PLIN5 interactions were found to be more spatially responsive to forskolin stimulation, compared with ABHD5-PLIN5 interactions that were exclusively found at the lipid droplet and were not dependent on S155 phosphorylation. This infers that while a large proportion of PLIN5 interacts with ABHD5, the PLIN5 phosphorylation state is not critical for their interaction and does not change during stimulated lipolytic conditions.

Recent work has shown that catecholamines induce PLIN5 translocation to the nucleus, where it enters a complex with SIRT1 and PGC-1α to induce transcription of genes that regulate mitochondrial biogenesis and oxidative metabolism (28, 29). Phosphorylation of PLIN5 was found to be both necessary and sufficient for nuclear translocation (28). In addition, ATGL and PLIN5 were reported to be co-obligatory to increase PGC-1α activity and that ATGL inhibition does not influence PKA-mediated translocation of PLIN5 to the nucleus (28, 29). Given that a large proportion of fatty acids that enter the cell first traverse the intracellular lipid droplet before their eventual oxidation (50), it is possible that PLIN5 conducts parallel roles of lipolysis and transcriptional activation to promote efficient fatty acid catabolism. We thereby assessed the impact of PKA activation and PLIN5 phosphorylation on nuclear PLIN5 abundance using live cell imaging. Consistent with previous studies, our data show that a proportion of PLIN5 colocates with the nucleus, that PLIN5 nuclear localization is increased with PKA stimulation, and that the forskolin-induced nuclear translocation is ablated in the PLIN5 S155A mutant. While PKA-induced PLIN5 nuclear translocation is dependent on phosphorylation of S155, the functional relevance remains unclear as basal and maximal mitochondrial respiration was not impaired in PLIN5 S155A cells. It is possible that sustained activation of PKA, which occurs during moderate to high intensity exercise training, might be required to unmask any PLIN5-mediated transcriptional regulation that would enhance mitochondrial function.

PLIN5 is localized to several intracellular locations including the lipid droplet, endoplasmic reticulum (51), mitochondria, and the nucleus (29), implying regulatory functions beyond lipolysis. Mitochondrial PLIN5 has attracted considerable attention because it may regulate the dynamic interaction between the lipid droplet and mitochondria to facilitate efficient transfer of fatty acid substrate for energy production (52), although this remains controversial (13, 53). In support of this possibility, PLIN5 has a mitochondrial binding domain positioned in the C terminus between amino acids 343 and 463 (52) and PLIN5 is targeted to the lipid droplet-mitochondria interface (31). Live cell imaging analysis in PLIN5 MEF cells showed that lipid droplet-mitochondria interaction increased more than 2-fold with PKA activation. This interaction was blunted considerably in the PLIN5 S155A mutant. In concordance with these findings, PLIN5 S155A mutants had reduced rates of triglyceride-derived fatty acid oxidation, despite normally functioning mitochondria, indicating that PKA-mediated phosphorylation of S155 on PLIN5 regulates mitochondrial fatty acid oxidation by coordinating fatty acid flux out of the lipid droplet and increasing lipid droplet-mitochondria contact. Future studies are required to determine whether PLIN5 acts as a *bone fide* organelle tethering protein.

The importance of PLIN5 serine phosphorylation in vivo was previously undetermined. Expression of PLIN5 and PLIN5 S155A in the livers of *Plin5*^*LKO*^ mice induced few overt phenotypic changes in mice, with similar body mass and composition, food intake and energy expenditure, respiratory exchange ratio, liver triglyceride content, liver triglyceride secretion, and circulating triglyceride concentrations between groups. Notably, PLIN5 S155 phosphorylation does not overtly impact these aspects of metabolism *in* *vivo*. The finding that liver triglyceride secretion was unaltered agrees with the previous studies showing that hepatic ATGL-derived lipolytic products do not provide a substrate for very low density lipoprotein secretion (54, 55).

Changes in intracellular lipid metabolism are intimately linked to insulin action (56, 57), and accelerated intracellular lipolysis that exceeds the requirements of cells for fatty acid oxidative metabolism can lead to the accumulation of lipid metabolites that cause insulin resistance and impair blood glucose control. Similar to our previous findings (36), administration of AAV-PLIN5 reversed the characteristic glucose intolerance inherent in *Plin5*^*LKO*^ mice. Interestingly, AAV-S155A mice exhibited a mild impairment in glucose tolerance, independent of changes in insulin sensitivity. An intriguing finding was the marked attenuation of glucose-induced insulin secretion in AAV-S155A mice, which is likely to contribute to the impaired glycemic control. The factors mediating this response are unknown but may result from changes in hepatokine secretion and hepato-pancreatic cross talk (58), changes in incretin signaling, or a generalized defect in β-cell function. Such possibilities will be the focus of future investigations.

In conclusion, these data provide *in* *vitro* and *in* *vivo* evidence that phosphorylation of S155 on PLIN5 is important for regulating intracellular lipolysis and triglyceride-derived fatty acid oxidation, through the combined actions of PLIN5-ATGL interactions at the surface of lipid droplets and by promoting lipid droplet-mitochondria interactions upon β-adrenergic stimulation. This work expands the evolving understanding of the carefully coordinated events controlling intracellular lipid metabolism.

## Materials and methods

### Expression and purification of murine Plin5

Total RNA was isolated from mouse adipose tissue using TRIzol reagent (Invitrogen, Carlsbad, CA) according to the manufacturer's protocol. RNA (3 μg) was reverse transcribed using the Thermoscript RT-PCR system (Invitrogen) and oligo dT primers. *Plin5* cDNA was PCR amplified using Platinum Taq (ThermoFisher Scientific, Melbourne, Australia) with the following primers: mouse *Plin5* (forward: GAC GGT ACC ATG GAC CAG AGA GGT GAA GAC ACC ACCC; reverse: TAG GAA TTC TCA GAA GTC CAG CTC TGG CAT TGTG). cDNA was cloned into a pcDNA3.1 vector (ThermoFisher Scientific) through BamHI and EcoRI sites. Murine *Plin5* underwent site-directed mutagenesis using pfx polymerase (Invitrogen) to generate serine to alanine substitutions as follows: Peptide S155A forward: CGG CGT TGG GCT GGG GAG CTG AGG CGC TCC, reverse: CAG CTC CCC AGC CCA ACG CCG GCC CCT TTGG, S161A forward: CTG AGG CGC GCC ATG AGT CAA GCC ATG GAC, reverse: CTT GAC TCA TGG CGC GCC TCA GCT CCC CAC, S163 forward: GCG CTC CAT GGC TCA AGC CAT GGA CAT GGTG, reverse: CAT GGC TTG AGC CAT GGA GCG CCT CAG CTCC.

### Sf21 cell culture, expression, and protein purification

The PCR product was subcloned into the pFASTBAC-FLAGTev vector (*Bam*HI/*Sal*I). Sf21 insect cells were grown in Sf-900 II medium at 27°C, with shaking at 130 rpm. Recombinant FLAG-PLIN5 baculovirus was plaque purified and amplified to high titer. For expression, cells were infected using a multiplicity of infection of 2 and were at 20% cell death 48–72 h after infection. Expressed protein was extracted from 480 ml of cell pellet on FLAG antibody resin (A2220, Sigma, Rowville, Australia).

Cells were harvested by centrifugation at 1,500 *g* for 15 min and the pellets sonicated for 3 × 30 s at setting 3 with a Branson probe sonicator (model 150, Fisher Scientific) in 40 ml of 50 mM HEPES buffer (pH 7.2), 150 mM NaCl, 1% Triton X-100, 1 mM EGTA, 10% glycerol, 2 mM phenylmethylsulfonylfluoride, 10 μg/ml leupeptin, 1 μg/ml pepstatin, and 0.01% sodium azide. The cell lysate was cleared by centrifugation at 20,000 *g* for 20 min and incubated with 2 ml of FLAG antibody resin 3.1 mg/ml overnight at 4°C. FLAG-PLIN5 was eluted with 0.25 mg/ml FLAG peptide (DYKDDDDK) in Tris-buffered saline, 10% glycerol, and 0.01% Triton X-100. The preparation was concentrated by ultrafiltration at 4°C on an Amicon 10 mL Ultra centrifugal filter to 2 mg/ml.

### Phosphorylation of recombinant PLIN5 in vitro

Recombinant murine PLIN5 (45 μM) was phosphorylated by cAMP-dependent protein kinase (PKA), catalytic subunit (P6000S, 250 U/reaction; Sigma, St. Louis, MO), containing 250 μM [γ-^32^P]ATP (5,000 cpm/pmol) for 2 h at 30°C. The protein mixture was separated by SDS-PAGE, transferred to a PVDF membrane, and the ^32^P-labeled PLIN5 was detected by phosphorimaging (ChemiDoc MP and ImageLab software Version 4.1, Bio-rad Laboratories, NSW, Australia). PLIN5 loading was confirmed by immunoblot analysis using anti-PLIN5 antibody (anti-MLDP/PLIN5, GP31, Santa Cruz).

### Mass spectrometry analysis of phosphopeptides

Proteins were excised from SDS-polyacrylamide gels, reduced with TCEP-HCl (Thermo Scientific), carbamidomethylated with iodoacetamide (Sigma), and digested with trypsin (Sigma). The tryptic peptides were extracted with 50% acetonitrile (acidified with 1% formic acid), lyophilized in a vacuum concentrator, and reconstituted in 15 μl buffer A (0.1% formic acid). Prior to mass spectrometry, the peptides were further purified and enriched using OMIX C18 Mini-Bed tips (Agilent Technologies, Mulgrave, Australia).

Using a Dionex UltiMate 3000 RSLCnano system equipped with a Dionex UltiMate 3000 RS autosampler, an Acclaim PepMap RSLC analytical column (75 μm × 50 cm, nanoViper, C18, 2 μm, 100 Å; Thermo Scientific), and an Acclaim PepMap 100 trap column (100 μm × 2 cm, nanoViper, C18, 5 μm, 100 Å; Thermo Scientific), the tryptic peptides were separated by increasing concentrations of 80% ACN/0.1% formic acid at a flow of 250 nl/min for 50 min and analyzed with a QExactive mass spectrometer (Thermo Scientific).

To obtain peptide and phosphopeptide sequence information, the raw files were searched with Andromeda implemented in MaxQuant v1.6.3.4 (59) against a focused database containing only Q8BVZ1 (Plin5) and P05132 (Prkaca) in addition to common contaminants. Carbamidomethylation at cysteine residues was specified as a fixed modification. Oxidation at methionine residues, N-terminal acetylation, and phosphorylation at serine, threonine, or tyrosine residues were set as variable modifications. Trypsin/P was selected as protease and up to three missed cleavages were permitted. Precursor mass tolerances were set to 20 ppm and 4.5 ppm on the first and main search, respectively, while fragment ions were searched with a 20 ppm mass tolerance. Only peptides identified within a false discovery rate of 1% based on a decoy database were further analyzed. The proteomics data have been deposited to the ProteomeXchange Consortium via the PRIDE partner repository with the data set PXD021969; (Username: reviewer_pxd021969@ebi.ac.uk; Password: 1O6XcChO) (60).

### Retroviral constructs

Wild-type and mutant murine *Plin5* were amplified and ligated into BamHI (R0136S, New England Biolabs) and EcoRI (R0101S, New England Biolabs, Notting Hill, Australia) (NEB)-digested pEGFP C1 vector (CLONTECH, Mountain View, CA) using a recombination method with a Gibson cloning kit (E2611, New England Biolabs). The green fluorescent protein (GFP)-*Plin5* inserts were amplified and subcloned into BamHI and EcoRI (NEB)-digested pBABE-puro vector (Cell Biolabs, Inc., San Diego, CA) to generate knock in GFP-*Plin5*, GFP-*Plin5* S155A, GFP-*Plin5* S161A, and GFP-*Plin5* S163 constructs to transfect into *Plin5*^*−/−*^ murine embryonic fibroblasts. The constructs were sequence verified using BigDye terminator mix (Applied Biosystems, Foster City, California).

### Isolation of murine embryonic fibroblasts from wild-type and Plin5^−/−^ mice

MEFs (E14) were derived from *Plin5*^−/−^ and *Plin5*^+/+^ mice (13) by digesting with 0.02% collagenase (Sigma) for 1 h at 37°C as described (22). The cells were washed and plated overnight in high-glucose Dulbecco Modified Eagle Medium (DMEM, Life Technologies, Australia), 10% fetal bovine serum (FBS, ThermoFisher Scientific), and 0.5% penicillin-streptomycin before transfection. Cells were used up to passage 3 for experiments.

### Transfection of retroviral plasmids and infection of MEF

Retroviral vectors containing wild-type *Plin5* and mutant inserts were transfected using Lipofectamine LTX reagent (15338500, Invitrogen) into HEK293T cells (American Type Culture Collection CRL-3216) as previously described (61). The viral supernatant was collected after 24 h and passed through a 0.45 μm filter (Merck Millipore, Germany). MEFs were transduced with viral supernatant containing 3.3 mg/ml Polybrene (Sigma) for 5 h at 37°C. The viral supernatant was removed, and the infected MEFs were selected using 2.5 μg/ml puromycin (Invitrogen). Noninfected cells died within 2 d and the infected cells proliferated. MEFs were then grown in high-glucose DMEM, 10% fetal bovine serum, and 0.5% penicillin-streptomycin.

### Mitochondria and lipid droplet staining and imaging protocol

As previously described (36), MEFs expressing PLIN5 or mutated PLIN5 were incubated with MitoTracker (Molecular Probes, Eugene, OR) diluted (2 μmol) in prewarmed media for 15 min. Cells were washed with PBS. BODIPY (ThermoFisher Scientific) (2 μg/ml) was applied to cells and incubated for 10 min. Cells were washed with PBS and viewed using time-lapse confocal microscopy (TCS SP8 confocal microscope, Leica). Lipid droplet quantification was measured using ImageJ software. Edges are detected and local intensity maxima are determined in the preprocessed image. Edge-defined particles were obtained using thresholding followed by hole filling and watershedding. An edge-defined particle sharing coordinates with a local maximum is then counted as a “true” lipid droplet. The areas of the segmented particles are measured, and the radius and corresponding volume are calculated assuming that the area describes a circle. To quantify the distance between lipid droplets and mitochondria over several time points, a ×40 lens was calibrated at 1 pixel = 0.01679 μm, i.e., 100 pixels = 1.1679 μm. An Euclidean pixel-distance map was derived with any MitoTracker signal above threshold set as distance zero. The maxima of lipid droplets were localized by their position on the Euclidean distance map, across three time points, to determine proximity time away from the mitochondria. For example, if the lipid droplet touches a mitochondrion, the lipid droplet is zero pixels away from a mitochondrion, thus for that time point, that lipid droplet will have a measurement of zero. Additionally, if the lipid droplet sits 10 pixels away from the nearest mitochondria, then that droplet gets assigned a value of 10 for that time point. Therefore, if a droplet spends three time points touching a mitochondria, two time points being two pixels away, and one time point being five pixels away, the lipid droplet will receive a value of (3 × 0) + (2 × 2) + (1 × 5) = 9 units of proximity time. This interaction was quantified as percentage of proximity time.

### Fatty acid metabolism

MEFs containing wild-type and mutant *Plin5* were grown to confluence, then lipid-loaded for 12 h in low-glucose DMEM containing 500 μM palmitate: oleate (2:1) conjugated to 2% fatty-acid-free BSA. “Pulse-chase” experiments were performed to determine lipid droplet fatty acid metabolism. MEFs were “pulsed” in low-glucose DMEM containing 500 μM oleate (1 μCi/ml [1–^14^C] oleate) conjugated to 1% BSA for 12 h. Cells were washed 3× with warm PBS to remove all extracellular ^14^C-oleate. Some cells were lysed as described above and the content of ^14^C-oleate in triglyceride was determined. Adjacent cells were then incubated for 12 h in 1–^14^C oleate (i.e., “chased”) and 20 μM forskolin. At the completion of the experiment, the culture medium was added to 1 mL 1 M HClO_4_ to liberate ^14^CO_2_ derived from complete fatty acid oxidation, which was collected in 300 μL 1 M NaOH and counted on a Tri Carb 2810TR liquid scintillation analyzer (Perkin Elmer, Waltham, MA). Cells were washed 3× in ice-cold PBS, then scraped with PBS containing 0.1% Triton X-100, and passed through an insulin syringe. The cell lysate was added to 2:1 chloroform: methanol (v:v) and incubated for 2 h with intermittent mixing. The mixture was centrifuged at 200 *g* for 10 min and the upper aqueous phase was removed and counted by liquid scintillation to determine incomplete fatty acid oxidation. The lower organic phase was transferred to a fresh tube, dried under N_2_ at 40°C, then reconstituted in 2:1 chloroform: methanol containing lipid standards for triglyceride, diglyceride, and palmitate (Sigma). The lipid mixture was spotted onto a glass-backed Silica Gel 60 plate and the lipids were resolved. The plates were air-dried, sprayed with dichlorofluorescein (0.02% w/v in ethanol) dye, and the lipid bands were visualized under UV light. The lipid bands were scraped, and radioactivity incorporation assessed by lipid scintillation counting. Total fatty acid uptake was calculated by adding fatty acid oxidation to fatty acid incorporation into all cellular lipids. These values are taken to represent triglyceride-derived fatty acid oxidation. All values were normalized to total cellular protein (BCA method, ThermoFisher Scientific).

### Immunoblotting

Liver lysates were prepared in RIPA buffer, proteins were resolved by SDS-PAGE electrophoresis, and immunoblot analysis was conducted as described previously (62). Stain-free images were collected after transfer to correct for loading differences across samples (ChemiDoc MP and ImageLab software Version 4.1, Bio-rad Laboratories, Gladesville, Australia). The membranes were probed with antibodies raised against PLIN5 (GP31, Progen Biotechnik, Heidelberg, Germany) or actin (ab3280, Abcam). Data are presented as the density of the immunoreactive band relative to total protein loading for that specific lane.

### Assessment of mitochondrial respiration

Cells were plated at a density of 25,000 cells/well in Seahorse XFe24 cell culture microplates (Aligent) and placed in an incubator at 37°C for 60 min in culture media before experiments. All experiments were performed using an XFe24 Extracellular Flux Analyzer (Seahorse Bioscience, Lexington, MA) according to previously described methods, with slight modifications (63). Basal oxygen consumption rate (OCR; pmol/min) was determined in prewarmed Seahorse XF assay medium (Seahorse Bioscience) at ∼37°C (pH 7.4) supplemented with 25 mM glucose, 1 mM sodium pyruvate, and 1 mM glutamine. Seahorse injection ports were loaded with oligomycin (2 μM), carbonyl-trifluoromethoxy-phenylhydrazone (FCCP, 2 μM), rotenone (3 μM) and antimycin A (3 μM), giving final concentrations of final concentrations of 0.30, 0.26, and 0.35 μM, respectively. The Seahorse Analyzer was run using 9-min cyclic protocol commands (mix for 3 min, stand for 3 min, and measure for 3 min) in quadruplicate. Data was pooled to give average values and was normalized to protein content.

### Animal studies

Animal studies were approved by the Monash University School of Biomedical Science Animal Ethics Committee and conformed to the National Health and Medical Research Council of Australia guidelines regarding care and use of experimental animals. Liver-specific *Plin5* knockout mice (*Plin5*^*LKO*^) were generated as described previously (36) and were housed at 22°C on a 12:12-h light dark cycle. Wt and *Plin5*^*LKO*^ mice aged 8 weeks were fed a high-fat diet (43% energy from fat, Specialty Feeds, Glen Forrest, WA, Australia) for 12 weeks. An adeno-associated virus serotype 8 (AAV) driven by an albumin promotor, containing PLIN5 (AAV-PLIN5) or PLIN5-S155A (AAV-S155A) cDNA, was injected via the tail vein of *Plin5*^*LKO*^ mice (1 × 10^12^ gc/mouse). Mice had free access to water and were fasted for 4 h (0700–1100 h) before all experiments, which commenced at 1100 h unless otherwise stated. Male mice were used for all experiments.

### Determination of energy expenditure

Whole-body energy expenditure, the respiratory exchange ratio, food intake, and physical activity were assessed using a LabMaster calorimetry system (TSE Systems, Bad Homburg, Germany). Studies were commenced after 12 h of acclimation to the metabolic chamber. Expired gases were assessed at 30 min intervals for 48 h.

### Glucose and insulin tolerance tests and blood chemistry

Mice received an oral gavage of D-glucose (2 g/kg body mass) or intraperitoneal injection of insulin (1 U/kg body mass, Actrapid) for glucose and insulin tolerance tests, respectively. Blood obtained from a tail nick was assessed for glucose (Accu-Chek, Victoria, Australia) before and throughout the tests as indicated. Additional blood was obtained before and 15 and 30 min after glucose administration. The blood was spun (2,500 *g*, 5 min, 4°C) and the plasma used for later analysis of plasma insulin by ELISA (#90080, Crystal Chem, Elk Grove Village, IL). In separate experiments, blood was obtained from 4 h fasted mice for the determination of plasma triglycerides using the TG-GPO-PAP reagent (04657594190, Roche Diagnostics, Basel, Switzerland), plasma FFA (439-1750, Wako Chemicals, Wako, VA), and plasma β-hydroxybutyrate (MAK041, Sigma).

### Assessment of fatty acid metabolism in liver slices

Twelve weeks after the administration of AAVs, mice were fasted for 4 h. Mice were anaesthetized with 5% isoflurane in an induction chamber and sedation was maintained with 2% isoflurane in oxygen (8323001, Univentor, Malta). The liver was dissected, and the right lateral lobe was embedded in 3% agarose using the Tissue Embedding unit (Alabama Research and Development, Munford, AL) and sliced on the Alabama R&D Tissue Slicer (Alabama Research and Development) in 500 mL of warmed, pregassed (95% O_2_ / 5% CO_2_) Phenol Red Free DMEM with 1% P/S at 300 μm. Slices were collected, washed in Dulbecco's PBS, ∼20 mg liver slice was plated per well and settled in oxygenated (95% O_2_/5% CO_2_), prewarmed M199 media for 1 h. Subsequently, the tissue was placed in a glass vial and preincubated with 1 ml of warmed, pregassed (95% O_2_/5% CO_2_) M199 media supplemented with 10% fetal bovine serum (FBS, ThermoFisher Scientific, Australia) and 1% penicillin streptomycin (PenStrep) (ThermoFisher Scientific, Australia). Metabolism was then assessed in tissue slices as described for MEFs.

### Microscopy used for FRET

HeLa cells were grown in DMEM (Lonza, Basel, Switzerland) supplemented with 10% bovine growth serum (Gibco) and 1× Pen-Strep (Lonza) at 37°C in 5% CO_2_. For FLIM-FRET experiments, the HeLa cells were plated 24 h before experiments onto 35 mm glass bottom dishes and transiently transfected with the following plasmids via use of Lipofectamine 3000 according to the manufacturer's protocol: ATGL-CFP or ABHD5-CFP with PLIN5-YFP or PLIN5 S155A-YFP. These plasmids were obtained from previously published work (17, 23, 33). All FLIM-FRET data was acquired with an Olympus FV3000 laser scanning microscope coupled to a 440 nm pulsed laser operated at 20 MHz and an ISS A320 FastFLIM box for time-resolved detection. A 60 X water immersion objective 1.2 NA was used for all experiments and the cells were imaged at 37°C in 5% CO_2_. A 440 nm dichroic mirror was used to separate the fluorescence signal from the laser light. The fluorescence signal was then directed through a 518 nm long pass filter that split the donor and acceptor signal between two photomultiplier detectors (H7422P-40 of Hamamatsu) fitted with the following bandwidth filters: CFP 488/50 and YFP 520/25. The pixel frame size was set to 256, which gave a pixel size of 104 μm. The pixel dwell time was set at 20 μs / pixel, which for a 256- pixel frame size resulted in a 1.61 s frame time. For each FLIM experiment, 20 frames were integrated. Together these conditions resulted in an acquisition time of ∼0.5 min. Calibration of the system and phasor plot space was performed by measuring fluorescein (pH 9.0), which has a known single exponential lifetime of 4.04 ns. The FLIM data were acquired by ISS Vista Vision and processed by the SimFCS software developed at the Laboratory for Fluorescence Dynamics (LFD) (www.lfd.uci.edu).

### FLIM-FRET analysis

FLIM-FRET data was quantified by the phasor approach to fluorescence lifetime analysis (34, 35, 64). In brief, the fluorescence decay recorded in each pixel of a FLIM-FRET image is described by a *g* and *s* coordinate (phasor) in the phasor plot that when used in reciprocal mode, enables each point of the phasor plot to be mapped to each pixel of the FLIM image. In the case of a FRET experiment where the lifetime of the donor molecule is changed upon interaction with an acceptor molecule, the realization of all possible phasors quenched with different efficiencies describes a curved trajectory in the phasor plot. As described in previous papers (34, 35, 64), the FRET trajectory follows the classical definition of FRET efficiency and the contribution of the background (cellular autofluorescence) versus donor without acceptor (unquenched donor), both of which are determined independently, are evaluated using the rule of the linear combination. Briefly, acquisition of a FLIM image in the donor channel records the fluorescence lifetime in each pixel, producing a readout of FRET and protein-protein interaction. In particular, those pixels with a quenched lifetime undergo FRET. We therefore quantify the fraction of pixels that exhibit this quenched lifetime as a percentage. Additionally, we quantified the FRET efficiency of this interaction. This is related to how quenched the fluorescence lifetime is and produces a readout of the affinity of the protein-protein interaction.

The FRET efficiency of each donor-acceptor interaction was first characterized and then this phasor location was used to spatially map ATGL versus ABHD5 interaction with PLIN5, as well as quantify the frequency (*i.e.*, fraction of pixels) of this interaction across multiple cells. In a subanalysis, this workflow was restricted to only lipid droplets by application of an intensity threshold that selected this highly intense intracellular compartment. To increase phasor accuracy a 3 × 3 spatial median filter was applied to the FLIM maps presented in the result sections prior to FRET analysis. All FLIM-FRET quantitation was performed in the SimFCS software developed at the LFD.

### Statistics

All data are presented as mean ± SEM. In comparisons made between two groups, an unpaired two-tailed Student's *t* test was performed. In comparisons with more than two groups, one-way or two-way analysis of variance (ANOVA) was performed with Bonferroni post hoc analysis where appropriate. The analysis used for specific experiments is outlined in the figure legends. Statistical significance was established a priori at *P* < 0.05.

### Data availability

The authors declare that all data is contained in the manuscript. The proteomics data have been deposited to the ProteomeXchange Consortium via the PRIDE partner repository with the dataset PXD021969 (Username: reviewer_pxd021969@ebi.ac.uk; Password: 1O6XcChO).

## Conflict of interest

The authors declare that they have no conflicts of interest with the contents of this article. This work has not been submitted for publication elsewhere. M. J. W. is the guarantor of this work and, as such, had full access to all the data in the study and takes responsibility for the integrity of the data and the accuracy of the data analysis.
